# Bone Marrow-Derived Mesenchymal Stem Cells Repair Necrotic Pancreatic Tissue and Promote Angiogenesis by Secreting Cellular Growth Factors Involved in the SDF-1***α***/CXCR4 Axis in Rats

**DOI:** 10.1155/2015/306836

**Published:** 2015-02-25

**Authors:** Daohai Qian, Jian Gong, Zhigang He, Jie Hua, Shengping Lin, Chenglei Xu, Hongbo Meng, Zhenshun Song

**Affiliations:** Department of General Surgery, Shanghai Tenth People's Hospital, Tongji University of Medicine, Shanghai 200072, China

## Abstract

Acute pancreatitis (AP), a common acute abdominal disease, 10%–20% of which can evolve into severe acute pancreatitis (SAP), is of significant morbidity and mortality. Bone marrow-derived mesenchymal stem cells (BMSCs) have been reported to have a potential therapeutic role on SAP, but the specific mechanism is unclear. Therefore, we conducted this experiment to shed light on the probable mechanism. We validated that SDF-1*α* significantly stimulated the expressions of VEGF, ANG-1, HGF, TGF-*β*, and CXCR4 in BMSCs, which were inhibited by its receptor agonist, AMD3100. The capacities of proliferation, migration, and repair of human umbilical vein endothelial cells were enhanced by BMSCs supernatant. Meanwhile, BMSCs supernatant could also promote angiogenesis, especially after the stimulation with SDF-1*α*. *In vivo*, the migration of BMSCs was regulated by SDF-1*α*/CXCR4 axis. Moreover, transplanted BMSCs could significantly alleviate SAP, reduce the systematic inflammation (TNF-*α↓*, IL-*1β↓,* IL-6↓, IL-4↑, IL-10↑, and TGF-*β↑)*, and promote tissue repair and angiogenesis (VEGF↑, ANG-1↑, HGF↑, TGF-*β↑*, and CD31↑), compared with the SAP and anti-CXCR4 groups. Taken together, the results showed that BMSCs ameliorated SAP and the SDF-1*α*/CXCR4 axis was involved in the repair and regeneration process.

## 1. Introduction

Though acute pancreatitis (AP) is considered one of the commonest acute abdominal diseases, no effective treatment has yet been available. The incidence of AP is rising continually with 10%–20% of patients progressing to severe acute pancreatitis (SAP) [1], which is associated with significant morbidity and mortality. AP can also transform into chronic pancreatitis and even pancreatic cancer and the overall mortality rate is reported to be about 15%–20% [2]. However, the pathogenic mechanisms of AP have still not been understood. It is well recognized that AP begins with pancreatic acinar injury, attributed to the premature activation of trypsin within the pancreatic acinar cells, after which exudation, edema, and a local inflammatory response are observed [3, 4]. Further pathological deterioration can be prevented by the self-regulation of the bodies in most patients. But some AP patients can still progress to SAP with hemorrhage, necrosis, and a systematic inflammatory response, further leading to shock, multiple organ failure, and even death. Consequently, the treatment of SAP focuses predominantly on inhibiting the synthesis and secretion of trypsin and averting the systemic inflammatory response. Most patients with SAP require drug treatment, whereas some choose to receive surgery. However, no satisfactory therapeutic effect has been discovered whichever method is adopted.

Mesenchymal stem cells (MSCs), first described by Friedenstein et al. in 1976 [5], are multipotent adult stem cells that can be derived from many different organs and tissues. MSCs are characterized by the expression of cell-surface molecules, including CD44, CD73, CD90, and CD105 [6]. MSCs can differentiate into bone, cartilage, muscle, adipose tissue, and so forth* in vitro*, depending on the culture conditions [7]. MSCs also have low or no immunogenicity which make them ideal choices for cell transplantation. Many studies have also demonstrated that MSCs have therapeutic effects on several autoimmune, ischemic, and inflammatory diseases [8]. Therefore, they are widely valued and studied. MSCs have been isolated initially from bone marrow and much of the literature uses these cells [9–11], though other sources of MSCs have been described [12]. Recent researches have also shown that bone marrow-derived mesenchymal stem cells MSCs (BMSCs) are involved in the pathogenesis of AP and can relieve the severity and improve the prognosis of SAP [9–11]. Thus, we selected these cells as our therapeutic tool for SAP. Jung et al. [9] found that BMSCs reduced the levels of proinflammatory cytokines and increased the numbers of Foxp3+ regulatory T cells to improve SAP repair. Tu et al. [10] believed that BMSCs attenuated systemic inflammation through relieving injury to pancreatic acinar cells and small intestinal epithelium. However, the specific mechanism has been a controversy so far. In our previous work, we have firstly demonstrated that stromal-cell-derived factor 1*α* (SDF-1*α*) and its receptor, chemokine receptor 4(CXCR4), played an important role in the process of BMSCs therapy for SAP [11].

SDF-1*α* is a chemokine that regulates the migration of BMSCs by interacting with CXCR4 [13, 14]. However, recent studies have also shown that the SDF-1*α*/CXCR4 axis not only regulates the migration of cells, but also repairs damaged tissue and promotes angiogenesis [15, 16]. SDF-1*α*/CXCR4 axis has been reported to induce neovascularization in ischemic, tumor, and wounded tissues [17–19]. Besides, SDF-1*α* combines with vascular endothelial growth factor (VEGF) to enhance angiogenesis [20, 21]. Therefore, we come to the conclusion that the SDF-1*α*/CXCR4 axis is probably involved in the repair of acute necrotizing pancreatitis through promoting the formation of new vessels.

To demonstrate our hypothesis, we conducted this experiment, further investigating whether SDF-1*α*/CXCR4 axis can regulate the migration of BMSCs to injured pancreas and validating whether it can also enhance tissue repair and regeneration to recover SAP by inducing angiogenesis in rats.

In the current study, we used superparamagnetic iron oxide nanoparticles (SPION) to label BMSCs in order to track their distribution, as previously described [22, 23]. SPION have frequently been used to trace BMSCs* in vivo*. Recent studies have also indicated that the proliferation, migration, and differentiation capacities of SPION-labeled BMSCs are not affected by labeling (maintaining their “stemness”) [24, 25].

## 2. Materials and Methods

### 2.1. Materials and Reagents

Sodium taurocholate, CXCR4 agonist (AMD3100), dimethyl sulfoxide (DMSO), 3-(4,5-dimethylthiazol-2-yl)-2, S-diphenyltetrazolium bromide (MTT), 4′,6-diamidino-2-phenylindole (DAPI), poly-l-lysine, and Trypan Blue were purchased from Sigma-Aldrich (Brooklyn, NY, USA). The amylase activity assay kit was from Biovision (Palo Alto, California, USA), SPION (Fe_2_O_3_, 30 nm) were from Dk Nanotechnology Co. Ltd (Beijing City, China), Lipofectamine 2000, Dulbecco's modified Eagle's medium–low glucose (DMEM-LG), penicillin, streptomycin, fetal bovine serum (FBS), and the Histostain-Plus Kit (DAB, Broad Spectrum) were from Invitrogen (Carlsbad, California, USA), 0.25% trypsin was from Hyclone (Logan, Utah, USA), Transwell plates were from Corning (NY, USA), BCA protein concentration assay kit, RIPA lysis buffer, TRIzol Reagent, phenylmethanesulfonyl fluoride (PMSF, 100 mM), and the Prussian blue staining kit were from Beyotime Biotechnology Research Institute (Nantong City, Jiangsu Province, China), recombinant rat SDF-1*α* protein was from Peprotech (Rocky Hill, USA), Matrigel matrix was from Becton, Dickinson and Company (New Jersey, USA), Antibodies directed against CXCR4 were from Abcam (Cambridge, MA, USA), and VEGF, angiopoietin-1 (ANG-1), and GAPDH, and a fluorescent secondary antibody were from ProteinTech (Wuhan City, Hubei Province, China). Enzyme-linked immunosorbent assay (ELISA) kits for interleukin 1*β* (IL-1*β*), IL-4, IL-6, IL-10, tumor necrosis factor *α* (TNF-*α*), and transforming growth factor *β* (TGF-*β*) were purchased from R&D Systems (Minneapolis, MN, USA).

### 2.2. Animal Model

Healthy Sprague Dawley rats weighing 200–250 g (*n* = 99) were purchased from Shanghai Laboratory Animal Co. Ltd (Shanghai, China). All animals were maintained at about 25°C with an alternating 12 h dark/12 h light cycle, with a free access to standard laboratory water and food. An animal model of SAP was established by retrograde pancreatic duct injection of Na-taurocholate as previously described [14, 26]. All animal procedures were conducted according to the Shanghai Laboratory Animal Ordinance and approved by the Ethics Committee of Shanghai Tenth People's Hospital (Tongji University, Shanghai, China).

### 2.3. Cells and Cell Culture

BMSCs were isolated, cultured, and identified as described in our previous study [14]. A human umbilical-vein endothelial cell line (EA.hy926 cells) was purchased from Shanghai Cell Bank of the Chinese Academy of Sciences. EA.hy926 cells were cultured in DMEM-LG complete medium with 10% FBS, 100 U/mL penicillin and 100 *μ*g/mL streptomycin at 37°C in 5% CO_2_. The cells were digested and passaged by 1 : 3 when they reached >90% confluence.

### 2.4. Experimental Groups

Sixteenth-six rats were randomly divided into four groups: normal control (NC) (*n* = 6), sham (*n* = 6), SAP (*n* = 18), and SAP+BMSCs (*n* = 18), SAP+anti-CXCR4 BMSCs (*n* = 18). We established three subgroups among the SAP, SAP+BMSCs, and SAP+anti-CXCR4 BMSCs groups: SAP d1 (*n* = 6), d4 (*n* = 6), and d7 (*n* = 6), SAP+BMSCs d1 (*n* = 6), d4 (*n* = 6), and d7 (*n* = 6), and SAP+anti-CXCR4 BMSCs d1 (*n* = 6), d4 (*n* = 6), and d7 (*n* = 6). Another thirty-three rats were randomly divided into three groups to assess the distributions of SPION-labeled BMSCs: normal control (*n* = 3), BMSCs d1 (*n* = 3), d3 (*n* = 3), d5 (*n* = 3), d7 (*n* = 3), and d10 (*n* = 3) and anti-CXCR4 BMSCs d1 (*n* = 3), d3 (*n* = 3), d5 (*n* = 3), d7 (*n* = 3), and d10 (*n* = 3). BMSCs or SPION-labeled BMSCs (1 × 10^7^ cells/mL/kg) were injected into the rats through the tail vein for cell therapy or tracing. All animals were killed with a lethal dose of pentobarbital injected into the abdominal cavity. Recombinant SDF-1*α* protein (10 ng/mL or 100 ng/mL) was directly added to the BMSCS culture medium* in vitro*. Alternatively, BMSCs were stimulated with SDF-1*α* after culture with AMD3100 (10 *μ*g/mL) for 1 h. EA.hy926 cells in 25 cm^2^ flasks were first damaged by the addition of 100 *μ*L of 0.25% trypsin and then incubated in DMEM-LG complete medium supplemented with or without BMSCs supernatant containing DMEM-LG complete medium and BMSCs secretions. To assess the migration capacity of the cells in response to BMSCs, EA.hy926 cells were cultured in the upper chamber of a Transwell apparatus and lower chamber was filled with DMEM-LG complete medium with or without BMSCs supernatant.

### 2.5. Sample Collection

Blood samples and pancreatic tissues were collected from the normal control, sham, SAP, and SAP+BMSCs, SAP+anti-CXCR4 BMSCs groups. The serum was first extracted by centrifugation at 8000 ×g at 4°C and then stored at −80°C. The pancreatic tissues were stored in liquid nitrogen or fixed in 4% paraformaldehyde. After pretreatment with SDF-1*α* for 48 h, the BMSCs supernatants were collected, including the normal control, SDF-1*α* 10 ng/mL, and SDF-1*α* 100 ng/mL groups, and stored at −80°C. Meanwhile, the normal BMSCs supernatants were also collected at 24 h after cells were given fresh DMEM-LG complete medium.

### 2.6. Hematoxylin-Eosin (H&E) Staining and the Detection of Serum Amylase Activity

H&E staining and the detection of serum amylase activity were performed as previously described [11].

### 2.7. ELISAs

The serum levels of IL-1*β*, IL-4, IL-6, IL-10, TNF-*α*, and TGF-*β* were analyzed with ELISAs. Samples of serum (150 *μ*L) were diluted with standard diluent (1 : 1), and then 40 *μ*L diluted samples were added to the test sample wells. The test samples (10 *μ*L) were added to the wells (a final fivefold dilution of the samples), without touching the well walls as far as possible, and gently mixed. The plate was closed with the closure plate membrane and incubated for 30 min at 37°C. Wash solution was diluted 30-fold (or 20-fold) with distilled water and reserved. The closure plate membrane was removed, the liquid in the wells was discarded, the wells were dried by swinging the plate, and washing buffer was added to every well. The plate was left to stand for 30 s and then drained, this was repeated five times, and the plate dried by patting. Horseradish peroxidase- (HRP-) conjugated reagent (Agrisera, Sweden) (50 *μ*L) was added to each well, except the blank control well. After the plate was closed with the plate closure membrane, it was incubated for 30 min at 37°C. Wash solution was diluted 30-fold (or 20-fold) with distilled water and reserved. Chromogen solution A (50 *μ*L) and chromogen solution B (50 *μ*L) were added to each well, and the plate was incubated for 10 min at 37°C in the dark. Stop solution (50 *μ*L) was added to each well to stop the reaction (the solution changed from blue to yellow). Taking the blank well as zero, the absorbance of each well was read at 450 nm 15 min after the stop solution was added. The concentrations of the rat inflammatory cytokines in the samples were determined by comparing the absorbance of the samples with standard curves. Each sample included three repeated measurements.

### 2.8. Immunohistochemistry

The Histostain-Plus kit was used for the immunohistochemical analysis. Paraffin-embedded pancreatic tissues were dewaxed and rehydrated. Hydrogen peroxide (3%) was used to inactivate any endogenous peroxidase for 20 min at room temperature. Antigen retrieval was performed in a high-pressure cooker for 30 min. The pancreatic slices were incubated overnight with rabbit anti-rat VEGF (1 : 50) at 4°C in a wet box, after the endogenous antigens were blocked with 5% bovine serum albumin (BSA) for 30 min at room temperature. On the second day, the slices were incubated with biotin-labeled secondary antibody (goat anti-rabbit, Epitomics, China) for 30 min at 37°C and then with HRP-conjugated streptavidin for 20 min at 37°C. After the slices were stained with diaminobenzidine and hematoxylin, they were dehydrated, cleared, and mounted with neutral resin. CD31 and vWF proteins were detected with a similar procedure.

### 2.9. Immunoblotting Assay

Each sample of pancreatic tissue (approximately 100 *μ*g) was crushed to power and 500 *μ*L of RIPA lysis buffer containing 5 *μ*L of PMSF (100 : 1) was added to extract the total proteins. The concentration of the total proteins was determined with the BCA method. The proteins (20 *μ*g) were separated with 12% SDS-PAGE and transferred to 0.45 *μ*m nitrocellulose membrane. After the membrane was blocked with 5% skim milk for 1 h, it was incubated overnight with anti-VEGF polyclonal antibody (1 : 1000) at 4°C. On the following day, the membrane was washed three times with phosphate-buffered saline (PBS) containing Tween 20 for 10 min each time and then incubated with a secondary goat anti-rabbit antibody (1 : 1000) at 37°C for 1 h and washed again three times. The experiment was repeated five times. Finally, the signal was detected with the Odyssey 3.0 analysis software (LI-COR Biotechnology, USA).

### 2.10. qRT-PCR

Total RNA was extracted from the cells and frozen pancreatic specimens with TRIzol Reagent. First-strand cDNA was synthesized with the PrimeScript Reverse Transcriptase Reagent Kit (Takara Biotechnology, Japan) with oligo(dT) and random primer. The gene expression of the BMSCs and EA.hy926 cells was quantified with the KAPA Kit (Kapa Biosystems, USA) and 50 ng of cDNA was amplified in a 10 *μ*L reaction using the Applied Biosystems 7500 Real-Time PCR system based on SYBR Green dye (Applied Biosystems). The primers were purchased from Beijing Genomics Institute (Beijing, China). Rat glyceraldehyde-phosphate dehydrogenase (GAPDH) was used as the endogenous control. The sequences of the primers are listed in Table 1. qRT-PCR was performed with the following cycling conditions: 95°C for 3 min, followed by 40 cycles of 95°C for 1 s and 60°C for 20 s. Quadruplicate cycle threshold (CT) values were analyzed with the SDS software (Applied Biosystems, USA), using the comparative CT method. The procedure was replicated three times. Each measurement was set three repeats.

### 2.11. Immunofluorescent Staining

Immunofluorescent staining was used to assess CXCR4 expression in SPION-labeled BMSCs, as previously described [11].

### 2.12. Labeling BMSCs

BMSCs at >90% confluence were pretreated with or without AMD3100 (10 *μ*g/mL) and then incubated with labeling medium containing 25 *μ*g/mL Fe^3+^ and 0.75 ng/mL poly-l-lysine for 24 h. After they were labeled, the cells were washed three times with PBS and harvested with 0.25% trypsin-EDTA. The viability of the cells was assessed with Trypan Blue exclusion before cell transplantation. The functions of the different concentrations of Fe^3+^-labeled BMSCs were analyzed in the following experiments.

### 2.13. Cell Migration Assay

In this experiment, 5 × 10^4^ EA.hy926 cells were added to the upper chamber of the Transwell apparatus and supplemented with 180 *μ*L of DMEM and 20 *μ*L of 1% BSA, and the lower chamber was filled with 500 *μ*L of DMEM-LG complete medium with or without BMSCS supernatant (100 *μ*L). After incubation for 12 h, the upper chamber was removed and the cells were fixed with 4% paraformaldehyde for 30 min. The lower surface of the filter membrane was stained with 0.1% crystal violet for 10 min in the dark. After the membrane was thoroughly washed, it was observed with a digital camera and light microscope. The experiment was repeated three times. Finally, the crystal violet was dissolved in 300 *μ*L of 33% acetic acid and the absorbance of the solution was measured at 573 nm with an ELISA plate reader (Gene Company Limited, HK, China).

### 2.14. Cell Proliferation Assay (MTT Test)

To investigate whether BMSCs could repair the injured vascular endothelial cells, we designed this experiment. First, EA.hy926 cells were cultured in 25 cm^2^ culture flasks and then incubated with 100 *μ*L of 0.25% trypsin-EDTA for 60 min at a constant temperature of 37°C in a humidified atmosphere of 95% O_2_ supplemented with 5% CO_2_ until their confluence reached approximately 90%. Finally, the cells were collected and seeded into 96-well plates at 2000 cells/well. Each well contained DMEM-LG complete medium with or without BMSCs supernatant (DMEM-LG complete medium: BMSCs supernatant = 1 : 1). MTT solution (20 *μ*L of 5 mg/mL) was added daily for seven days (12 h, 36 h, 68 h, 92 h, 121 h, and 145 h). The medium was then removed and 150 *μ*L of DMSO was added to each well. The plates were then shaken slowly for 10 min. Absorbance was measured with an ELISA plate reader at 490 nm. This experiment was repeated three times. SPION-labeled BMSCs were also assayed with MTT test after treatment with different concentrations of Fe^3+^ (25 *μ*g/mL, 50 *μ*g/mL, 75 *μ*g/mL, or 100 *μ*g/mL).

### 2.15. ANG-1 and VEGF Silencing with Small Interfering RNAs

Gene silencing technology was used to understand the effects of the BMSCs supernatant on the angiogenic activity of EA.hy926 cells* in vitro*. Small interfering RNA (siRNA) targeting rat ANG-1 (5′-GGCCCAGAUACAACAGAAUUUdTdT-3′) and (5′-AUUCUGUUGUAUCUGGGCCUUdTdT-3′) or VEGF (5′-GGGATTGCACGGAAACUUUdTdT-3′) and (5′-AAAGUUUCCGUGCAAUCCCdTdT-3′) and control nonsilencing siRNAs (5′-AUGUGAAUGCACCAAAGAAdTdT-3′) and (5′-UUCUUUGGUCUGCAUUCACAUdTdT-3′) were synthesized by Biomics Biotechnologies Co., Ltd (Nantong City, Jiangsu Province, China). BMSCs were transfected with the siRNAs using Lipofectamine 2000, according to the manufacturer's protocol (Biotend, Shanghai, China). The BMSCs supernatant was collected 48 h after transfection and stored at −80°C for the tube formation assay.

### 2.16. Angiogenesis

A tube formation assay was performed to investigate the effect of the BMSCs supernatant on the angiogenic activity of EA.hy926 cells* in vitro*. Ninety-six-well culture dishes were coated with 50 *μ*L of matrigel matrix and incubated for 30 min at 37°C. EA.hy926 cells were starved for 24 h and were then seeded onto the solidified gels at a density of 10^5^ cells/well in 50 *μ*L of culture medium. The BMSCs supernatant (50 *μ*L) or 1 ×PBS (50 *μ*L) was added to each well. These cells were divided into the normal control, SDF-1*α* 10 ng/mL, SDF-1*α* 100 ng/mL, ANG-1 siRNA, and VEGF siRNA groups. After incubation for 6 h, the total tube-like structures were photographed with phase-contrast microscopy (100x) and five randomly selected microscopic fields per photograph were quantified using the Image J software. To further detect the angiogenesis* in vivo*, the expressions of vascular endothelial markers (VEGF, vWF, and CD31) were measured by immunohistochemistry or western blotting in SAP, SAP+BMSCs, and SAP+anti-CXCR4 BMSCs groups.

### 2.17. Magnetic Resonance Imaging (MRI) and Prussian Blue Staining

To track the distributions of SPION-labeled BMSCs* in vivo*, MRI and Prussian blue staining were used to detect Fe^3+^. The animals were anesthetized and then put into an MRI coil (Shanghai Niumag Electronic Science and Technology Co., Ltd, China). The T1-weighted imaging (T1WI) parameters were as follows: FOV read = 100 mm, FOV phase = 100 mm, TR = 500 ms, TE = 20 ms, slice width = 3.5 mm, slice gap = 0.5 mm, and NS = 12, and the T2-weighted imaging (T2WI) parameters were as follows: FOV read = 100 mm, FOV phase = 100 mm, TR = 2000 ms, TE = 80 ms, slice width = 3.5 mm, slice gap = 0.5 mm, and NS = 8. Finally, the animals were killed and pancreas, lung, liver, spleen, and small intestine were collected and fixed in 4% paraformaldehyde for Prussian blue staining. A Prussian blue staining kit was used to detect Fe^3+^ in the SPION-labeled BMSCs and above tissues, according to the manufacturer's instructions. The cells were fixed in 4% glutaraldehyde, washed, and incubated for 30 min with 2% potassium ferric-ferrocyanide (Perl's reagent) in 3.7% hydrochloric acid. The cells were washed again, counterstained with Nuclear Fast Red, and the iron staining was evaluated with light microscopy (Carl Zeiss, Oberkochen, Germany) with a ×40/0.75 objective lens and Axiovision 4.4 software (Carl Zeiss). The SPION labeling efficiency was determined by manually counting the Prussian blue stained and unstained cells under a Zeiss microscope (Carl Zeiss) at ×100 magnification with a ×100/1.30 oil-immersion objective lens. The percentage of labeled cells was determined as the average of five high-powered fields. After the slices were fixed, dehydrated, embedded, and sectioned, the tissues were stained with Prussian blue, as described above. The blue granules were counted by a person, who did not know the experiment, from ten areas of each histologic section.

### 2.18. Image Processing and Statistical Analysis

Adobe Photoshop 6.0 (Adobe Systems Inc., San Jose, CA), Image-Pro Plus version 6.0 (Media Cybernetics, USA), and Image J (National Institutes of Health, USA) were used for image typesetting, analysis, and processing. GraphPad Prism 5.0 (GraphPad Co., USA) was used for mapping. The SPSS 17.0 statistical software (Chicago, IL) was used for all statistical analyses. Experimental data are shown as means ± standard deviations (SD) and compared with Student's or a paired *t* test or one-way ANOVA. A value of *P* < 0.05 was deemed to indicate significant differences.

## 3. Results

### 3.1. Recombinant SDF-1*α* Protein Stimulated BMSCs Expressions of VEGF, ANG-1, HGF, TGF-*β*, and CXCR4

**Figure 1:**
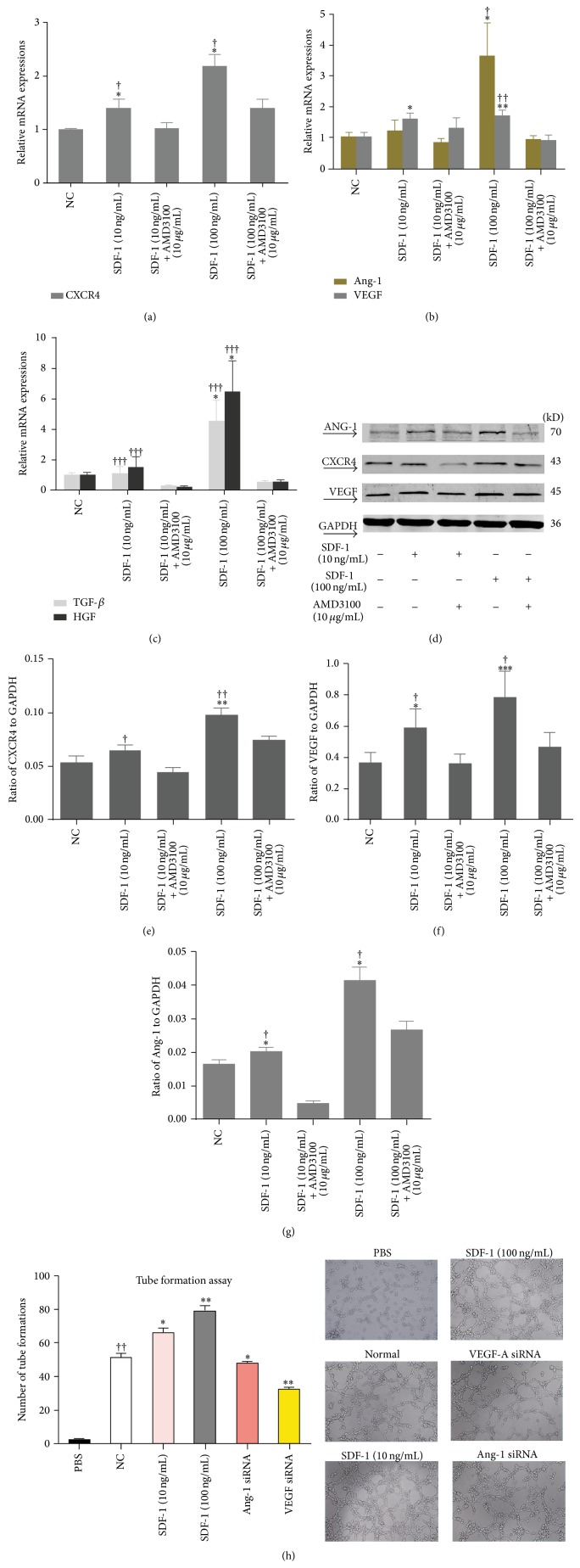
The SDF-1*α*/CXCR4 axis could promote the expressions of cellular growth factors in BMSCs and induce angiogenesis* in vitro*. ((a), (d), and (e)) The CXCR4 expression of BMSCs was significantly unregulated after being stimulated by combined SDF-1*α* protein, which could be inhibited by the CXCR4 receptor agonist, AMD3100. ((b)–(d), (f)-(g)) Recombined SDF-1*α* protein could significantly promote BMSCS expression of VEGF, ANG-1, HGF, and TGF-*β* and the effect could also blocked by AMD3100. Moreover, there was a dose-effect relationship between combined SDF-1*α* protein and CXCR4, VEGF, ANG-1, HGF, and TGF-*β* expressions. Data are expressed as mean ± SD (^*^ *P* < 0.05, ^**^ *P* < 0.01, and ^***^ *P* < 0.001 for SDF-1*α* (10 ng/mL or 100 ng/mL) versus NC, ^†^ *P* < 0.05, ^††^ *P* < 0.01, and ^†††^ *P* < 0.001 for SDF-1*α* (10 ng/mL or 100 ng/mL) versus SDF-1*α* (10 ng/mL or 100 ng/mL) + AMD3100 10 *μ*g/mL). (h) BMSCs supernatant could significantly promoted angiogenesis, especially after being pretreated with SDF-1*α* and this increase was partly offset by AMD3100. Both VEGF siRNA and Ang-1 siRNA significantly weakened proangiogenic capacity of BMSCs. Data are expressed as mean ± SD (^##^ *P* < 0.01 for NC versus PBS, ^*^ *P* < 0.05, ^**^ *P* < 0.01, and ^***^ *P* < 0.001 for SDF-1*α* (10 ng/mL or 100 ng/mL), Ang-1 siRNA, or VEGF siRNA versus NC) (NC, normal control, SDF-1*α*, stromal cell derived factor-1a, CXCR4, CXC chemokine receptor 4, VEGF, vascular endothelial growth factor, and ANG-1, angiopoietin-1).

The expressions of VEGF, ANG-1, HGF, TGF-*β*, and CXCR4 were detected with qRT-PCR or immunoblotting assays at 24 h and 48 h after recombinant SDF-1*α* protein was added to the BMSCs culture medium. The recombined SDF-1*α* protein significantly stimulated the expressions of VEGF, ANG-1, and CXCR4 in the BMSCs, which was also positively correlated with the concentration of SDF-1*α*. This effect was blocked by the CXCR4 agonist, AMD3100 (Figures 1(a)-1(b) and 1(d)–1(g)). The mRNA levels of HGF and TGF-*β* were both upregulated after stimulation with SDF-1*α* but were inhibited by AMD3100 (Figure 1(c)).

### 3.2. BMSCs Supernatant Promotes the Proliferation, Migration, and Repair of EA.hy926 Cells

**Figure 2:**
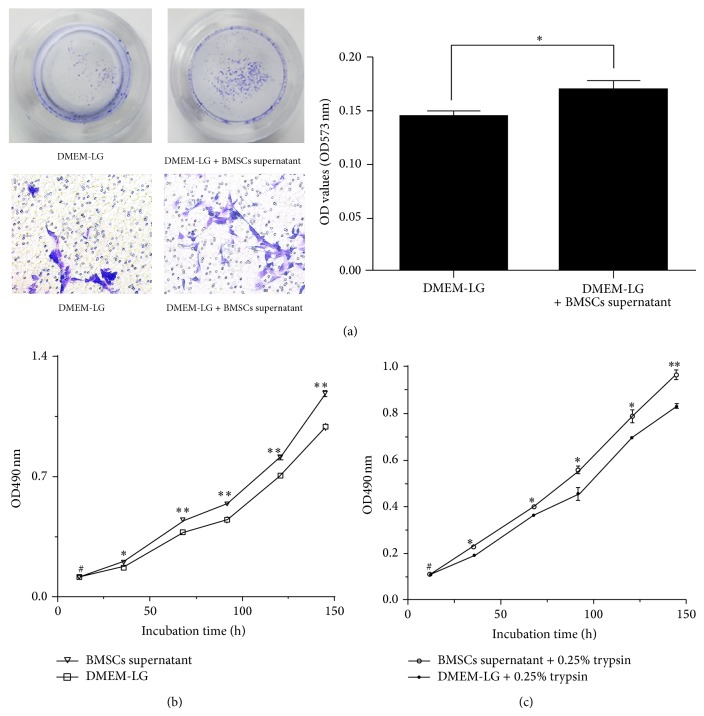
The BMSCs supernatant could promote the proliferation, migration, and repair of human umbilical-vein endothelial cell line (EA.hy926 cells). (a) The methylene blue staining (magnification, ×200) is indicating that the BMSCs supernatant could significantly promote the migration of EA.hy926 cells. Data are expressed as mean ± SD (^*^ *P* < 0.05 for DMEM-LG versus DMEM-LG + BMSCs supernatant). (b) The significant increase of the growth of EA.hy926 cells was investigated in MTT test after being given BMSCs supernatant. (c) Impaired EA.hy926 cells could be repaired by BMSCs supernatant. Data are expressed as mean ± SD (^#^ *P* > 0.05 for sham versus normal, ^*^ *P* < 0.05 and ^**^ *P* < 0.01 for DMEM-LG versus DMEM-LG + BMSCs supernatant at each corresponding time point). Data analysis was performed by a one-way ANOVA. (BMSCs, bone marrow-derived mesenchymal stem cells, MTT, 3-(4,5-dimethylthiazol-2-yl)-2,S-diphenyltetrazolium bromide).

The BMSCs supernatant promoted the growth and migration of EA.hy926 cells significantly more than DMEM-LG complete medium (Figures 2(a) and 2(b)). Moreover, EA.hy926 cells impaired by trypsin were more effectively repaired by the BMSCs supernatant than by DMEM-LG complete medium (Figure 2(c)).

### 3.3. SDF-1*α*/CXCR4 Axis Induces Angiogenesis* In Vitro,* Being Related to the Expressions of VEGF and ANG-1

The BMSCs supernatant pretreated with SDF-1*α* significantly promoted angiogenesis compared with the normal BMSCs supernatant* in vitro*, which was also positively related to the concentration of SDF-1*α*. Furthermore, the angiogenesis was reduced in both VEGF siRNA and ANG-1 siRNA compared with normal BMSCs supernatant (Figure 1(h)).

### 3.4. No Significant Differences on Biological Functions between BMSCs and SPION-Labeled BMSCs ([Fe^3+^] = 25 *μ*g/mL)

**Figure 3:**
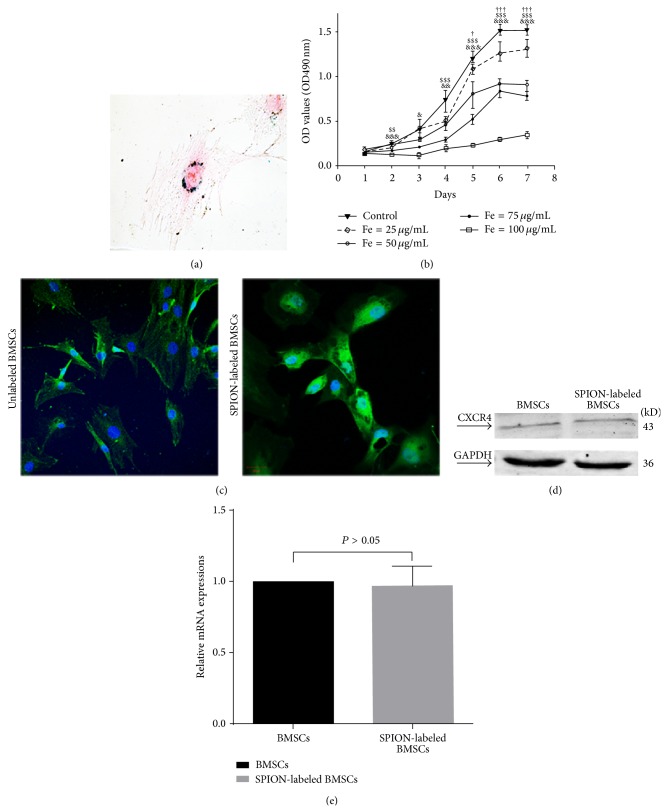
There is similarity in the proliferation and CXCR4 expression between unlabeled and SPION-labeled BMSCs ([Fe^3+^] = 25 g/mL). (a) The Prussian blue staining shows that BMSCs were labeled by SPION successfully. (b) The MTT tests are showing that the growth activity of SPION-labeled BMSCs ([Fe^3+^] = 25 g/mL) slowed slightly compared with that of the unlabeled BMSCs, which was also significantly stronger than that of other SPION-labeled BMSCs ([Fe^3+^] = 50, 75, or 100 g/mL). Data are expressed as mean ± SD (^&&^ *P* < 0.01 and ^&&&^ *P* < 0.001 for normal control versus SPION-labeled BMSCS ([Fe^3+^] = 100 g/mL), ^ $$^ *P* < 0.01 and ^ $$$^ *P* < 0.001 for normal control versus SPION-labeled BMSCS ([Fe^3+^] = 75 g/mL), and ^†^ *P* < 0.05 and ^†††^ *P* < 0.001 for normal control versus SPION-labeled BMSCS ([Fe^3+^] = 50 g/mL) at each corresponding time point). ((c)-(d)) CXCR4 expression was detected by immunofluorescence and western blotting and similar between unlabeled and SPION-labeled BMSCs (*P* > 0.05). (SPION, superparamagnetic iron oxide nanoparticles, BMSCs, bone marrow-derived mesenchymal stem cells).

More than 95% of BMSCs were successfully labeled with SPION ([Fe^3+^] = 25 *μ*g/mL) (Figure 3(a)). The growth activity of SPION-labeled BMSCs ([Fe^3+^] = 25 *μ*g/mL) was similar to unlabeled BMSCs, which was significantly stronger than that of other SPION-labeled BMSCs ([Fe^3+^] = 50, 75, or 100 *μ*g/mL) (Figure 3(b)). CXCR4 expression was observed with confocal microscopy and similar in both the BMSCs and SPION-labeled BMSCs (Figures 3(c)–3(e)).

### 3.5. SDF-1*α*/CXCR4 Axis Regulates the Migration of SPION-Labeled BMSCs to Necrotic Pancreatic Tissues and the Migration Increased Gradually, Peaking on Days 5–7

**Figure 4:**
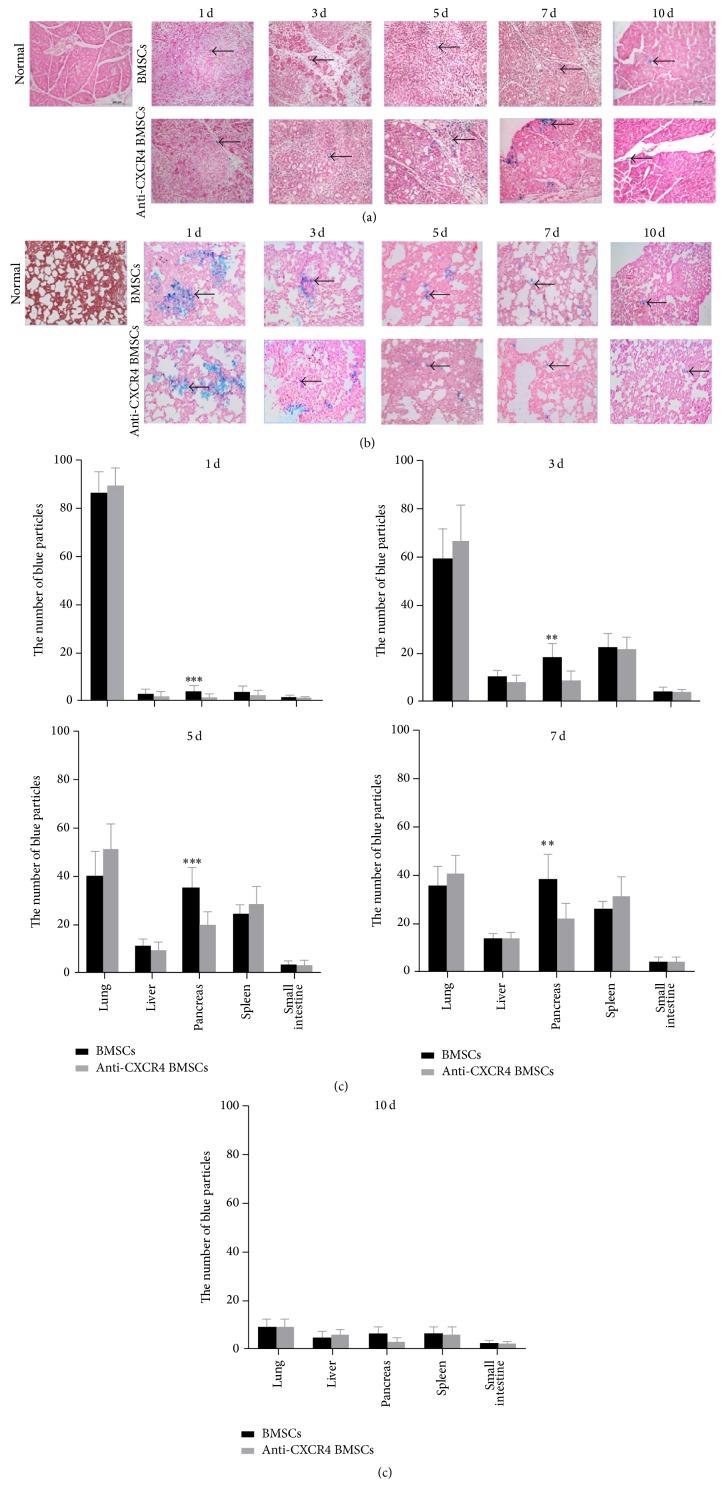
Prussian blue staining was performed for detecting the SPION-labeled BMSCs in pancreatic and lung tissues. (a) The Prussian blue staining of pancreatic tissue indicates that the cells were stained blue (as indicated by black arrows), gradually increased, and peaked on postoperative days 5–7, when the formation of tubular complexes was also maximal (as indicated by red arrows). However, the migration was partly inhibited and the trend was not obviously investigated in anti-CXCR4 group. (b) The lung tissues were stained by Prussian blue and the blue particles (as indicated by black arrows) were gradually decreasing in both BMSCs and anti-CXCR4 groups. (c) The blue particles in lung, pancreas, liver, spleen, and small intestine were counted and analyzed between BMSCs and anti-CXCR4 groups. The result showed that the number of blue particles of pancreas in BMSCs group was significantly more than in anti-CXCR4 group at postoperative days 1, 3, 5, and 7. Data are expressed as mean ± SD (^**^ *P* < 0.01 and ^***^ *P* < 0.001 for BMSCs versus anti-CXCR4 at each corresponding time point).

**Figure 5:**
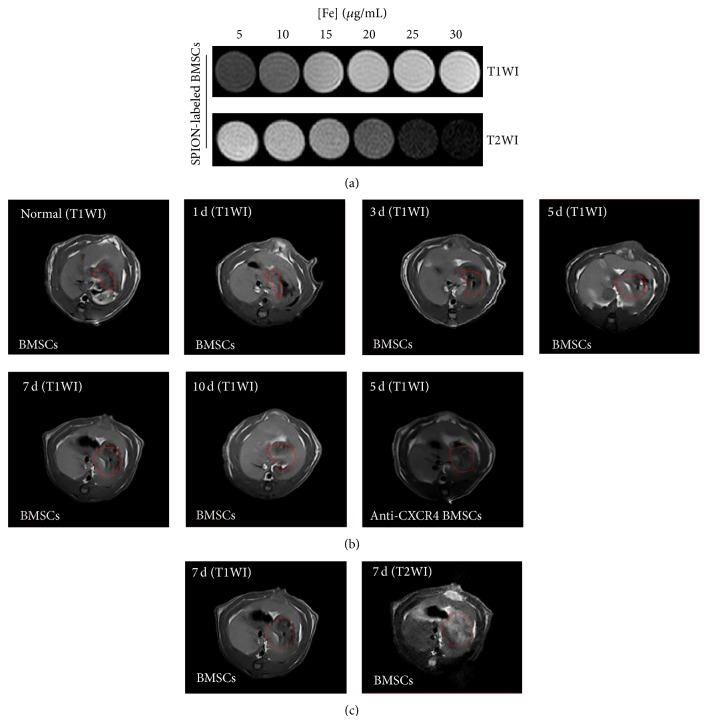
The transplanted SPION-labeled BMSCs were tracked by MRI* in vivo*. (a) [Fe^3+^] was detected by MRI and the result showed that the SPION-labeled BMSCs ([Fe^3+^] = 25 g/mL) appeared high signals (white spots) on T1WI and low signals (dark spots) on T2WI. (b) The SPION-BMSCs ([Fe^3+^] = 25 g/mL) was tracked by MRI* in vivo* and the photos show that the white spots (inside of red circle) were increasing gradually and peaked on postoperative days 5–7. Instead, the white spots decreased on postoperative day 5 in anti-CXCR4 group compared with BMSCs group. (c) The SPION-BMSCs ([Fe^3+^] = 25 g/mL) were detected in SAP rats at postoperative day 7 by T1WI and T2WI and the result was showing that the high signals (white spots), which appeared in T1WI, became low signals (dark spots) in T2WI (SPION, superparamagnetic iron oxide nanoparticles, T1WI, T1-weighted imaging, and T2WI, T2-weighted imaging).

The results of Prussian blue staining showed that the SPION-labeled BMSCs were relatively low in pancreas, liver, spleen, and small intestine at the first day of the transplant compared to lung, but gradually increasing in pancreatic tissue at posttransplant days 3 and 7 and relatively high compared to liver, spleen, and small intestine, when the formation of tubular complexes (TCs) was also maximal (Figures 4(a)–4(c)). On the contrary, SPION-labeled BMSCs in the lung tissue became less and less (Figure 5(b)). The more important point was that the migrations of SPION-labeled BMSCs to injured pancreas could be inhibited by anti-CXCR4 treatment (Figures 4(a)–4(c)). To further visually investigate whether the SPION-labeled BMSCs could migrate to injured pancreas, the MRI method was selected for realizing the tracing* in vivo*. The magnetic resonance images displayed that SPION-labeled BMSCs ([Fe^3+^] = 25 g/mL) were high signal in T1WI (white) and low signal (dark) in T2WI as shown in Figure 5(a). The high signal intensity (white points) in pancreas (as indicated by red circle) increased gradually in T1WI and peaked on days 5 and 7 (Figure 5(b)). We also performed T2WI on day 7 in order to exclude the possibility of false positive and the result showed that the high signals (white points) in T1WI became low signals (dark points) in T2WI, whereas the low signals (dark points) in T1WI became high signals (white points) as shown in Figure 5(c). Furthermore, the high signals in T1WI decreased obviously in anti-CXCR4 group compared with BMSCs group on posttransplant day 5 (Figure 5(b)).

### 3.6. Transplanted BMSCs Reduced Pancreatic Edema, Hemorrhage, and Necrosis, Inhibited Systematic Inflammation, and Promoted the Formation of TCs Involved in the SDF-1*α*/CXCR4 Axis

**Figure 6:**
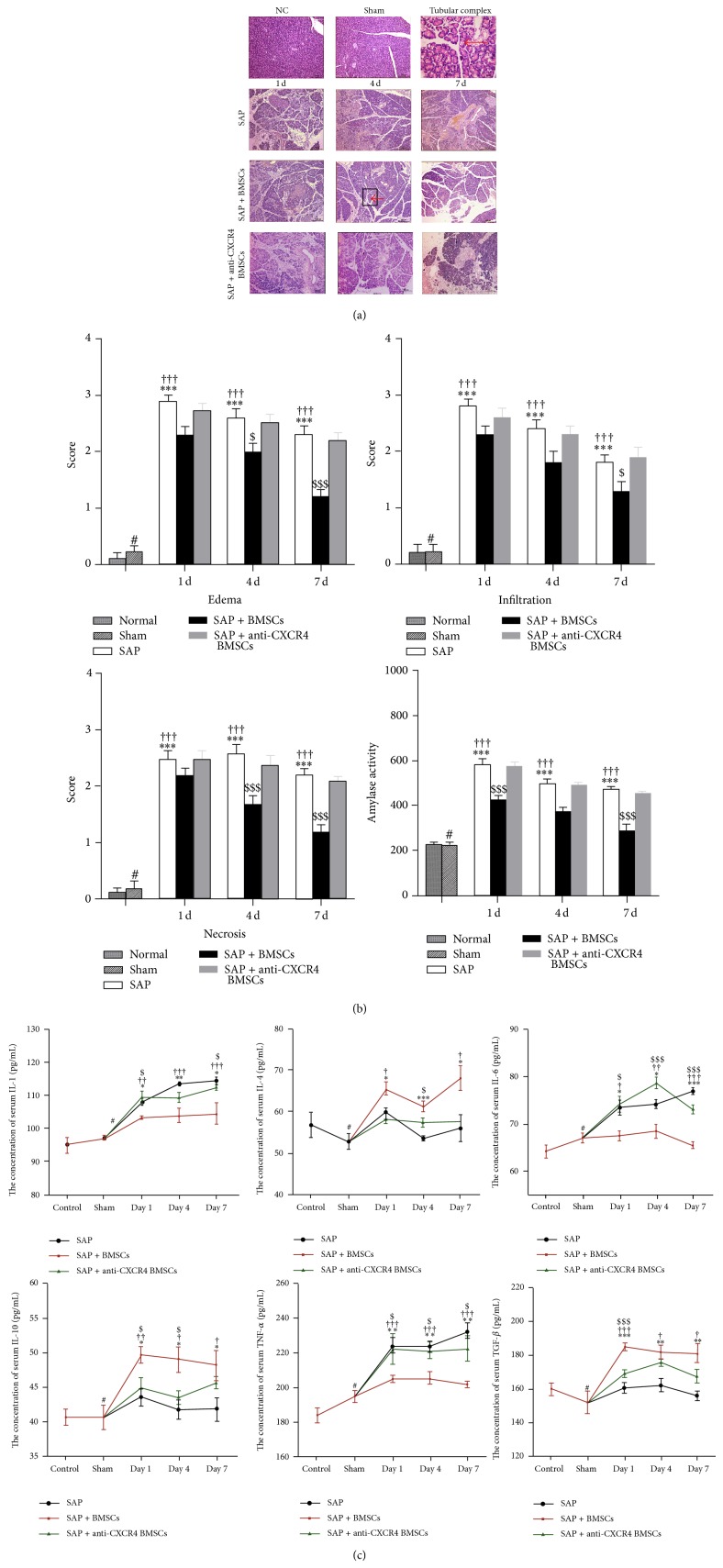
Transplanted BMSCs could reduce severe acute pancreatitis (SAP), promoting the formation of tubular complexes and inhibiting systematic inflammatory response, being involved in the SDF-1*α*/CXCR4 axis. ((a), (b)) The H&E staining of pancreatic tissues (magnification, ×100) is showing that the edema, infiltration, and necrosis were significantly reduced in the SAP+BMSCs group compared with SAP and SAP+anti-CXCR4 BMSCs groups at postoperative days 1, 4, and 7, respectively. A large number of tubular complexes were also investigated in SAP+BMSCs group (as indicated by red arrow in a 400x magnified picture). Serum amylase activity was also significantly reduced in SAP+BMSCs group than in normal, SAP, and anti-CXCR4 BMSCs groups at postoperative days 1, 4, and 7, respectively. (c) The levels of serum proinflammatory cytokines are significantly lower in SAP+BMSCs than in SAP and SAP+anti-CXCR4 BMSCs groups. In contrast, the levels of serum anti-inflammatory cytokines are significantly higher in SAP+BMSCs than in SAP and SAP+anti-CXCR4 BMSCs groups. Data are expressed as mean ± SD (^#^ *P* > 0.05 for sham versus normal, ^*^ *P* < 0.05, ^**^ *P* < 0.01, and ^***^ *P* < 0.001 for SAP versus Normal, ^†^ *P* < 0.05, ^††^ *P* < 0.01, and ^†††^ *P* < 0.001 for SAP versus SAP+BMSCs, ^ $^ *P* < 0.05, ^ $$^ *P* < 0.01, and ^ $$$^ *P* < 0.001 for SAP+BMSCs versus SAP+anti-CXCR4 BMSCs). Data analysis was performed by Student's test (BMSCs, bone marrow-derived mesenchymal stem cells, H&E, hematoxylin-eosin).

Pancreatic edema, hemorrhage, and necrosis were markedly reduced and the levels of serum amylase were significantly lower in the SAP+BMSCs group than in the SAP and SAP+anti-CXCR4 BMSCs groups (Figures 6(a) and 6(b)). Inflammatory cytokines were detected with ELISAs. The results show that the levels of serum proinflammatory cytokines (IL-1*β*, IL-6, and TNF-*α*) were significantly downregulated in the SAP+BMSCs group compared with the SAP and SAP+anti-CXCR4 BMSCs groups. In contrast, the levels of serum anti-inflammatory cytokines (IL-4, IL-10, and TGF-*β*) were significantly upregulated in the SAP+BMSCs group compared with the SAP and SAP+anti-CXCR4 BMSCs groups (Figure 6(c)). We also found that a large number of TCs appeared in the BMSCs group (as indicated by red arrow and a magnified picture in Figure 6(a)).

### 3.7. SDF-1*α*/CXCR4 Axis Enhances the Expressions of VEGF and ANG-1 in Damaged Pancreatic Tissues

**Figure 7:**
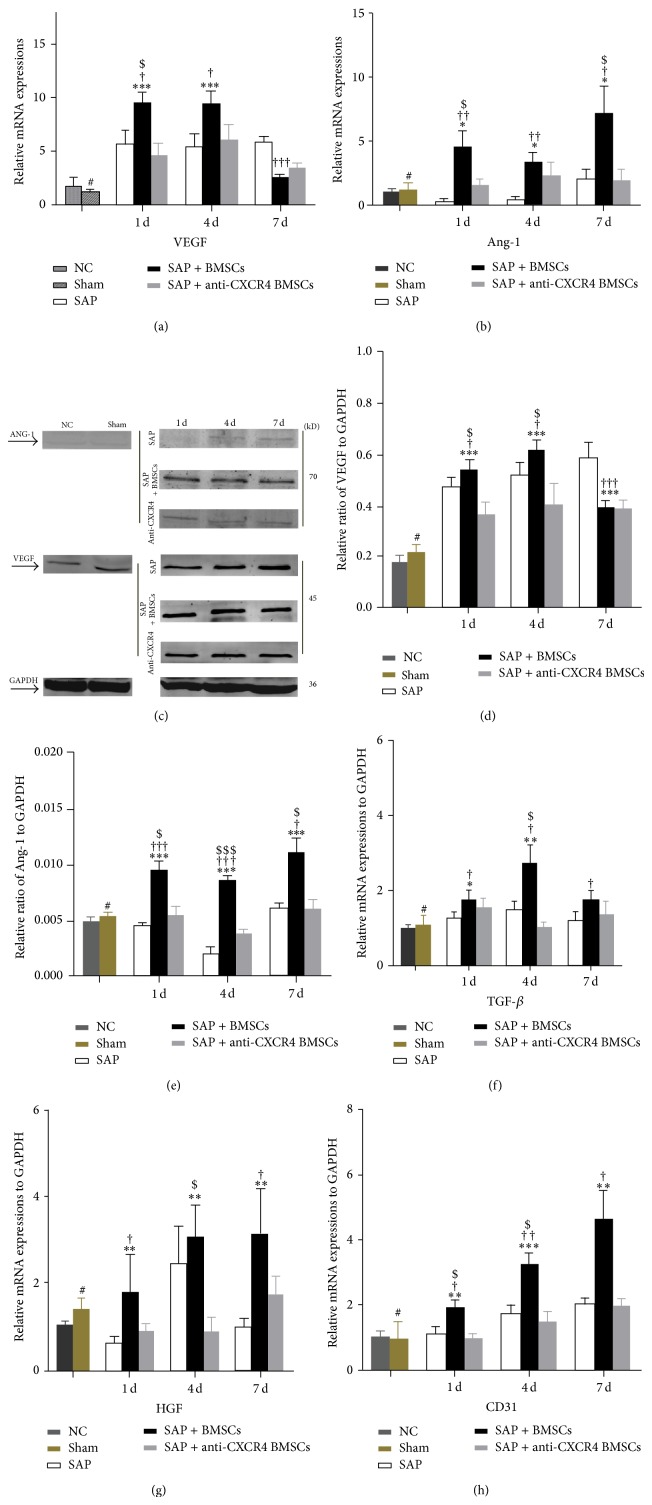
qRT-PCR or western-blot assays are showing that the expressions of ANG-1, VEGF, HGF, TGF-*β*, and CD31 in pancreatic tissue were significantly higher in SAP+BMSCs group. ((a), (c), and (d)) The expression of ANG-1 was investigated higher in SAP+BMSCs group than in normal, SAP, and SAP+anti-CXCR4 BMSCs groups. ((b), (c), and (e)) There was a significant higher expression of VEGF in SAP+BMSCs group than in normal, SAP, and SAP+anti-CXCR4 BMSCs groups at posttransplant days 1 and 4, with a subsequent decrease at day 7. ((f)–(h)) The mRNA level of HGF, TGF-*β*, and CD31 was significantly higher in SAP+BMSCs group than in normal, SAP, and SAP+anti-CXCR4 BMSCs groups. Data are expressed as mean ± SD (^#^ *P* > 0.05 for sham versus NC, ^*^ *P* < 0.05 and ^**^ *P* < 0.01 for SAP+BMSCs versus NC, ^†^ *P* < 0.05, ^††^ *P* < 0.01, and ^†††^ *P* < 0.001 for SAP+BMSCs versus SAP, ^ $^ *P* < 0.05, ^ $$^ *P* < 0.01, and ^ $$$^ *P* < 0.001 for SAP+BMSCs versus SAP+anti-CXCR4 BMSCs at each corresponding time point) (NC, normal control, ANG-1, angiopoietin-1, VEGF, vascular endothelial growth factor, SAP, severe acute pancreatitis, and BMSCs, bone marrow-derived mesenchymal stem cells).

VEGF expression in injured pancreatic tissues was measured with qRT-PCR and immunoblotting. VEGF expression was higher in the SAP+BMSCS group than in the normal control, SAP and SAP+anti-CXCR4 BMSCs groups on postoperative days 1 and 4 but had decreased on postoperative day 7 (Figures 7(b), 7(c), and 7(e)). The expression of ANG-1 was detected with qRT-PCR and immunoblotting and its expression was higher in the SAP+BMSCS group than in the normal control, SAP, and SAP+anti-CXCR4 BMSCs groups (Figures 7(a), 7(c), and 7(d)). The mRNA levels of HGF and TGF-*β* were also detected with qRT-PCR and were significantly higher in the SAP+BMSCs group than in the normal, SAP, and SAP+anti-CXCR4 BMSCs groups (Figures 7(f) and 7(g)).

### 3.8. SDF-1*α*/CXCR4 Axis Induces the Neovascularization* In Vivo*

**Figure 8:**
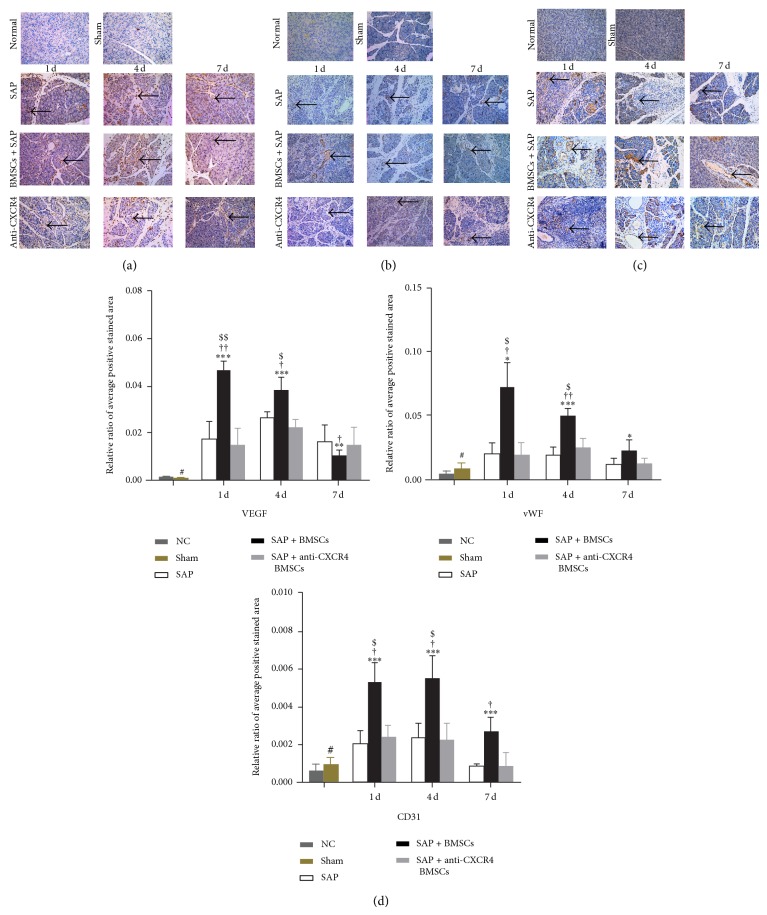
Transplantation BMSCs could promote angiogenesis in damaged pancreatic tissue, which was inhibited by anti-CXCR4 treatment. ((a), (d)) VEGF expression was assayed in pancreatic tissue by immunohistochemistry and the result showed that VEGF expression was higher in SAP+BMSCs group than in normal, SAP, and anti-CXCR4 groups at postoperative days 1 and 4. On the contrary, it was lower in SAP+BMSCs group than in SAP group at postoperative day 7. ((b)–(d)) vWf and CD31 expressions in pancreatic tissue were showed to be significantly higher in SAP+BMSCs group than in normal, SAP, and SAP+anti-CXCR4 BMSCs groups. Data are expressed as mean ± SD (^#^ *P* > 0.05 for sham versus NC, ^*^ *P* < 0.05 and ^**^ *P* < 0.01 for SAP+BMSCs versus NC, ^†^ *P* < 0.05, ^††^ *P* < 0.01, and ^†††^ *P* < 0.001 for SAP+BMSCs versus SAP, ^ $^ *P* < 0.05, ^ $$^ *P* < 0.01, and ^ $$$^ *P* < 0.001 for SAP+BMSCs versus SAP+anti-CXCR4 BMSCs at each corresponding time point) (NC, normal control, VEGF, vascular endothelial growth factor, SAP, severe acute pancreatitis, BMSCs, bone marrow-derived mesenchymal stem cells, vWF, von Willebrand factor, and CD31, platelet endothelial cell adhesion molecule-1).

To assess the neovascularization, we conducted qRT-PCR or immunohistochemistry for detecting these angiogenesis markers (CD31, VEGF, and vWF). The results indicated that the expressions of CD31, VEGF, and vWF in necrotic pancreatic tissue were higher in the SAP+BMSCs group than in the normal control, SAP, and SAP+anti-CXCR4 BMSCs groups, especially in early phase (Figures 7(h) and 8(a)–8(d)).

## 4. Discussion

Several studies have shown that MSCs from the bone or umbilical cord can attenuate acute necrotic pancreatitis by inhibiting systematic inflammation, regulating the immune responses, reducing the apoptosis of pancreatic acinar cells, and repairing damaged the small intestinal epithelium [9–12]. However, these functions do not account for how to repair and regenerate the necrotic pancreatic tissues. In the study, we found that BMSCs can reduce severe acute pancreatitis by alleviating the systematic inflammation, promoting the formation of tubular complexes (TCs), and inducing angiogenesis involving the SDF-1*α*/CXCR4 axis by enhancing the expression of cell growth factors in a rat model of SAP. Large previous studies have found that MSCs can not only differentiate into different kinds of functional adult cells but also secrete various soluble factors, including VEGF, HGF, and TGF-*β* [27]. These factors which promote angiogenesis and mitosis and reduce apoptosis can explain tissue repair and regeneration after MSCs transplanted [28, 29]. Moreover, the regeneration of the pancreas begins with the TCs that consist of a cluster of epithelia surrounded by the mesenchymal cells [30, 31]. Thus, we inferred that SDF-1*α*/CXCR4 axis was involved in the repair and regeneration of SAP.

SDF-1*α* and its receptor CXCR4 have been studied extensively, together with their roles in migration, homing, proliferation, angiogenesis, and so forth [11, 13–15, 18, 32, 33]. Many researches have shown that BMSCs can migrate to injured tissues* via* the SDF-1*α*/CXCR4 axis and relieve myocardial infraction [13, 14], promote wound healing [19], heal bone fracture [34], reduce cerebral ischemia [35], and ameliorate acute necrotizing pancreatitis [11]. SDF-1*α* expression is upregulated by hypoxia-inducible factor-1 (HIF-1) [36]. In the study, we successfully established a rat model of acute necrotizing pancreatitis with the retrograde injection of Na-taurocholate, which causes acinar cell injury/necrosis by triggering transient pathological intra-acinar-cell calcium ions and activating digestive zymogens [37–39]. The injured/necrotic pancreatic tissue causes HIF-1 expression and thus induces SDF-1*α* expression, which attracts BMSCs to injured pancreas through the interaction with CXCR4.

For further investigating whether BMSCs could migrate to injured pancreas and the migration was regulated by SDF-1*α*/CXCR4 axis in a SAP rat model, SPION was used to label BMSCs. SPION were initially used as a unique MR contrast agent in the clinical context. Recently, their two uses have been discovered—the labeling of live cells and tissues, disease diagnosis, and therapy [22, 23, 40–42]. Studies have demonstrated that SPION-labeling does not deleteriously affect the functions of the target cells [25]. In our study, we also demonstrated that there was no difference in the proliferation capacities between SPION-labeled (Fe = 25 *μ*g/mL, labeling efficiency > 95%) and unlabeled BMSCs. Moreover, the expression of CXCR4 was also similar between the labeled and unlabeled cells. SPION-labeled BMSCs could be detected by both MRI and Prussian blue staining because SPION is the nanometer particles of ferric oxide. The dynamic movement of labeling cells could be observed at the photos of MRI so that we can intuitively assess the proportions of SPION-labeled BMSCs* in vivo*. The result showed that these cells could migrate to the damaged pancreatic tissues and there was also a gradual increase in migration, peaking on postoperative days 5–7, with the formation of large numbers of TCs. SDF-1*α*/CXCR4 axis could regulate the migration of BMSCs as our previously described [11]. Meanwhile, the number of cells, which migrated to the necrotic pancreas, was positively related to the therapeutic effect. However, SPION-labeled BMSCs could be rarely seen on postoperative day 10, perhaps because the SPION were degraded or the BMSCs had differentiated into other cells, including pancreatic exocrine cells.

To validate whether the SDF-1*α*/CXCR4 axis also plays an important role in the repair and regeneration of SAP, we conducted the study* in vitro* and* vivo*. We found that the secretions of BMSCs could promote the proliferation, migration, and restoration of EA.hy926 cells. The BMSCs supernatant induced angiogenesis and the SDF-1*α*/CXCR4 axis stimulated BMSCs to express VEGF, ANG-1, HGF, and TGF-*β*, promoting further angiogenesis* in vitro*. Both VEGF and ANG-1 play important roles in angiogenesis. Moreover, the expressions of VEGF, ANG-1, HGF, and TGF-*β* in the damaged pancreatic tissues were higher in the BMSCs transplantation group than in the normal control, SAP, and SAP+anti-CXCR4 BMSCs groups. Taken all together, the result showed that transplanted BMSCs repaired the damaged pancreas through secreting large cellular growth factors and the effect was regulated by SDF-1*α*/CXCR4 axis.

As we all know, VEGF, a potent inducer of angiogenesis, promotes the repair of injured vascular endothelial cells and neovascularization. Studies have shown that the SDF-1*α*/CXCR4 axis promotes the synthesis and secretion of VEGF [17, 43] and SDF-1*α*, combined with VEGF, enhances ischemic angiogenesis [20]. In our study, we confirmed that the SDF-1*α*/CXCR4 axis correlates positively with VEGF expression in BMSCs.* In vivo*, VEGF expression in pancreatic tissues in the early phase of BMSCS transplantation was significantly higher than that in the normal, SAP, and SAP+anti-CXCR4 BMSCs groups. Therefore, we confirmed that the SDF-1*α*/CXCR4 axis is involved in the upregulation of VEGF expression in pancreatitis tissues. On the one hand, the upregulation of SDF-1*α* attracted BMSCs to damaged pancreatic tissues. On the other hand, the interaction of SDF-1*α* with CXCR4 increased the expression of VEGF, which repaired the damaged vascular endothelial cells and further enhanced angiogenesis. SDF-1*α* also increased the expression of the CXCR4 receptor as shown in our experiment and further promoted the migration of BMSCs to injured pancreatic tissues. Meanwhile, VEGF can also enhance the expression of CXCR4 [15]. Thus, the SDF-1*α*/CXCR4 axis and VEGF form a virtuous circle, producing a cascade effect. As a result, the association between the SDF-1*α*/CXCR4 axis and VEGF could account for the migration of BMSCs, the restoration of damaged vascular endothelial cells, neovascularization, and the ultimate repair and regeneration of necrotic pancreatic tissues. However, we also found that the expression of VEGF had decreased at posttransplant day 7 because the ischemia and hypoxia in the pancreatic tissues were significantly reduced after cell transplantation.

ANG-1, also involved in the process of angiogenesis, was upregulated by the SDF-1*α*/CXCR4 axis, and that ANG-1 expression was higher in the SAP+BMSCS group than in the normal, SAP, and SAP+anti-CXCR4 BMSCs groups. Large studies have also demonstrated that VEGF, combined with ANG-1, promotes angiogenesis more effectively [44]. Thus, the SDF-1*α*/CXCR4 axis, VEGF, and ANG-1 are jointly involved in the process of neovascularization of necrotic pancreatic tissues. In addition, HGF was significantly expressed after BMSCs were stimulated by the SDF-1*α*/CXCR4 axis. HGF expression was also elevated in damaged pancreatic tissues after BMSCS transplantation. HGF can stimulate epithelial cell proliferation, motility, morphogenesis, and angiogenesis in various organs* via* the tyrosine phosphorylation of its receptor, c-MET [45]. Bai et al. [46] found that HGF mediated MSC-induced recovery in a model of multiple sclerosis. Wang et al. [31] found that HGF/c-Met are expressed inside pancreatic tubular complexes and are possibly involved in the regeneration of islet cells. In our study, we have also confirmed that HGF participates in the process of repairing and regenerating necrotic pancreatic tissue.

As for TGF-*β*, it is a cytokine with a wide range of diversity and often has contradictory functions. Recent studies lay emphasis on its anti-inflammatory and antiatherogenic roles. TGF-*β* influences the activation of macrophages and T cells, as well as the proliferation of smooth muscle cells in the vessel wall [47, 48]. A reduction in the production or activity of this cytokine is believed to destabilize plaque. These and numerous other experimental data constitute the basis of David Grainger's TGF-*β* protective cytokine hypothesis [49, 50]. In our study, we found that the SDF-1*α*/CXCR4 axis also promotes the expression of TGF-*β in vitro* and that the TGF-*β* in both serum and pancreatic tissue was higher in the BMSCs+SAP group than in the normal, SAP, and SAP+Anti-CXCR4 groups* in vivo*. Therefore, TGF-*β* could also be involved in inhibiting inflammation and promoting the regeneration of damaged pancreas.

Many studies have shown that the systematic inflammatory response plays a key role in the deterioration of SAP. However, the inflammatory response is essential as a vascular reaction and could be relieved if the injured vessels could be repaired. Conversely, the repair and regeneration of injured vessels are helpful to promote tissue repair and regeneration. In the study, we found that damaged vascular endothelial cells were repaired and regenerated after BMSCs transplantation, which may be one of the reasons for the decrease of proinflammatory factors.

In conclusion, our results demonstrate that transplanted BMSCs reduce the systematic inflammatory responses, repair, and regenerate necrotic pancreatic tissues by secreting cytokines and promoting angiogenesis, with the involvement of the SDF-1*α*/CXCR4 axis, in a rat model of SAP. SPION is a good option for labeling BMSCs, and the SPION labeling method could be applied to live traces* in vivo*.

Of course, some problems have not yet been overcome in this study. There is still a certain distance from the animal research to the clinical application. Firstly, the necrotized pancreas was not fully repaired, especially in early phase. Secondly, the safety of BMSCs transplantation still needs to be tested. The occurrences of pulmonary embolism and venous thrombosis need to be further evaluated. Third,* in vivo*, the cell survival rate and time still need to be improved after BMSCs transplanted. Thus, the future research should be focused on how to lengthen cell survival time, enhance the functions of BMSCs, and improve the safety of BMSCs transplantation.

## Figures and Tables

**Table 1:** The primer sequences of genes.

Rat			
Gene	Forward primer	Reverse primer	PCR amplified products (bp)
GAPDH	5′>ACCACAGTCCATGCCATCAC<3′	5′>TCCACCACCCTGTTGCTGTA<3′	452
VEGF	5′>TGTGAGCCTTGTTCAGAGCG<3′	5′>GACGGTGACGATGGTGGTGT<3′	252
CXCR4	5′>TGTGAGCCTTGTTCAGAGCG<3′	5^'^>TGTGAGCCTTGTTCAGAGCG<3′	260
ANG-1	5′>CAAGGCTTGGTTACTCGTCAG<3′	5^'^>CCATGAGCTCCAGTTGTTGC<3′	109
HGF	5′>AGTCTATGGACCTGAAGGCTC<3′	5′>CCATCTGCGTTGATCAATCC<3′	171
TGF-*β*	5′>CTACTGCTTCAGCTCCACAGAG<3′	5′>CTGTACTGTGTGTCCAGGCTC<3′	162
CD31	5′>GAGTGCTTCTTGGTGGGTCC<3′	5′>CCTGAGAAGCACTGTACACC<3′	150

## Abbreviation

SDF-1*α*: Stromal-cell-derived factor-1*α*
VEGF: Vascular endothelial growth factor
ANG-1: Angiopoietin-1
HGF: Hepatocyte growth factor
TGF-*β*: Transforming growth factor-*β*
CXCR4: CXC chemokine receptor 4
TNF-*α*: Tumor necrosis factor-*α*
IL-1*β*: Interleukin-1*β*
CD31: Platelet endothelial cell adhesion molecule-1
vWF: von Willebrand factor
SPION: Superparamagnetic iron oxide nanoparticles
FOV: Field of vision
TR: Repetition time
TE: Echo time
NS: Number of scans.

## References

[1] Granger J., Remick D. (2005). Acute pancreatitis: models, markers, and mediators. *Shock*.

[2] Berezina T. L., Zaets S. B., Mole D. J., Spolarics Z., Deitch E. A., Machiedo G. W. (2005). Mesenteric lymph duct ligation decreases red blood cell alterations caused by acute pancreatitis. *The American Journal of Surgery*.

[3] Sah R. P., Garg P., Saluja A. K. (2012). Pathogenic mechanisms of acute pancreatitis. *Current Opinion in Gastroenterology*.

[4] Hirano T., Manabe T. (1993). A possible mechanism for gallstone pancreatitis: repeated short-term pancreaticobiliary duct obstruction with exocrine stimulation in rats. *Proceedings of the Society for Experimental Biology and Medicine*.

[5] Friedenstein A. J., Gorskaja J. F., Kulagina N. N. (1976). Fibroblast precursors in normal and irradiated mouse hematopoietic organs. *Experimental Hematology*.

[6] Pountos I., Corscadden D., Emery P., Giannoudis P. V. (2007). Mesenchymal stem cell tissue engineering: techniques for isolation, expansion and application. *Injury*.

[7] Herzog E. L., Chai L., Krause D. S. (2003). Plasticity of marrow-derived stem cells. *Blood*.

[8] Brooke G., Cook M., Blair C. (2007). Therapeutic applications of mesenchymal stromal cells. *Seminars in Cell and Developmental Biology*.

[9] Jung K. H., Song S. U., Yi T. (2011). Human bone marrow-derived clonal mesenchymal stem cells inhibit inflammation and reduce acute pancreatitis in rats. *Gastroenterology*.

[10] Tu X.-H., Song J.-X., Xue X.-J. (2012). Role of bone marrow-derived mesenchymal stem cells in a rat model of severe acute pancreatitis. *World Journal of Gastroenterology*.

[11] Gong J., Meng H.-B., Hua J. (2014). The SDF-1/CXCR4 axis regulates migration of transplanted bone marrow mesenchymal stem cells towards the pancreas in rats with acute pancreatitis. *Molecular Medicine Reports*.

[12] Yang B., Bai B., Liu C. X. (2013). Effect of umbilical cord mesenchymal stem cells on treatment of severe acute pancreatitis in rats. *Cytotherapy*.

[13] Yu J., Li M., Qu Z., Yan D., Li D., Ruan Q. (2010). SDF-1/CXCR4-mediated migration of transplanted bone marrow stromal cells toward areas of heart myocardial infarction through activation of PI3K/Akt. *Journal of Cardiovascular Pharmacology*.

[14] Theiss H. D., Vallaster M., Rischpler C. (2011). Dual stem cell therapy after myocardial infarction acts specifically by enhanced homing via the SDF-1/CXCR4 axis. *Stem Cell Research*.

[15] Salcedo R., Wasserman K., Young H. A. (1999). Vascular endothelial growth factor and basic fibroblast growth factor induce expression of CXCR4 on human endothelial cells. In vivo neovascularization induced by stromal-derived factor-1alpha. *American Journal of Pathology*.

[16] Du L., Yang P., Ge S. (2012). Stromal cell-derived factor-1 significantly induces proliferation, migration, and collagen type i expression in a human periodontal ligament stem cell subpopulation. *Journal of Periodontology*.

[17] Hiasa K.-I., Ishibashi M., Ohtani K. (2004). Gene transfer of stromal cell-derived factor-1*α* enhances ischemic vasculogenesis and angiogenesis via vascular endothelial growth factor/endothelial nitric oxide synthase-related pathway: next-generation chemokine therapy for therapeutic neovascularization. *Circulation*.

[18] Mukherjee D., Zhao J. (2013). The role of chemokine receptor CXCR4 in breast cancer metastasis. *The American Journal of Cancer Research*.

[19] Xu X., Zhu F., Zhang M. (2013). Stromal cell-derived factor-1 enhances wound healing through recruiting bone marrow-derived mesenchymal stem cells to the wound area and promoting neovascularization. *Cells Tissues Organs*.

[20] Yu J. X., Huang X. F., Lv W. M. (2009). Combination of stromal-derived factor-1*α* and vascular endothelial growth factor gene-modified endothelial progenitor cells is more effective for ischemic neovascularization. *Journal of Vascular Surgery*.

[21] Tang J.-M., Wang J.-N., Zhang L. (2011). VEGF/SDF-1 promotes cardiac stem cell mobilization and myocardial repair in the infarcted heart. *Cardiovascular Research*.

[22] Sun R., Dittrich J., Le-Huu M. (2005). Physical and biological characterization of superparamagnetic iron oxide- and ultrasmall superparamagnetic iron oxide-labeled cells: a comparison. *Investigative Radiology*.

[23] Pawelczyk E., Arbab A. S., Chaudhry A., Balakumaran A., Robey P. G., Frank J. A. (2008). In vitro model of bromodeoxyuridine or iron oxide nanoparticle uptake by activated macrophages from labeled stem cells: implications for cellular therapy. *Stem Cells*.

[24] Arbab A. S., Bashaw L. A., Miller B. R. (2003). Characterization of biophysical and metabolic properties of cells labeled with superparamagnetic iron oxide nanoparticles and transfection agent for cellular MR imaging. *Radiology*.

[25] Balakumaran A., Pawelczyk E., Ren J. (2010). Superparamagnetic iron oxide nanoparticles labeling of bone marrow stromal (Mesenchymal) cells does not affect their ‘sternness’. *PLoS ONE*.

[26] Perides G., van Acker G. J. D., Laukkarinen J. M., Steer M. L. (2010). Experimental acute biliary pancreatitis induced by retrograde infusion of bile acids into the mouse pancreatic duct. *Nature Protocols*.

[27] Mezey E. (2011). The therapeutic potential of bone marrow-derived stromal cells. *Journal of Cellular Biochemistry*.

[28] Amado L. C., Saliaris A. P., Schuleri K. H. (2005). Cardiac repair with intramyocardial injection of allogeneic mesenchymal stem cells after myocardial infarction. *Proceedings of the National Academy of Sciences of the United States of America*.

[29] Lin Y.-T., Chern Y., Shen C.-K. J. (2011). Human mesenchymal stem cells prolong survival and ameliorate motor deficit through trophic support in Huntington's disease mouse models. *PLoS ONE*.

[30] Reid L. E., Walker N. I. (1999). Acinar cell apoptosis and the origin of tubular complexes in caerulein-induced pancreatitis. *International Journal of Experimental Pathology*.

[31] Wang G.-S., Rosenberg L., Scott F. W. (2005). Tubular complexes as a source for islet neogenesis in the pancreas of diabetes-prone BB rats. *Laboratory Investigation*.

[32] Burger J. A., Kipps T. J. (2006). CXCR4: a key receptor in the crosstalk between tumor cells and their microenvironment. *Blood*.

[33] Bachelder R. E., Wendt M. A., Mercurio A. M. (2002). Vascular endothelial growth factor promotes breast carcinoma invasion in an autocrine manner by regulating the chemokine receptor CXCR4. *Cancer Research*.

[34] Kitaori T., Ito H., Schwarz E. M. (2009). Stromal cell-derived factor 1/CXCR4 signaling is critical for the recruitment of mesenchymal stem cells to the fracture site during skeletal repair in a mouse model. *Arthritis and Rheumatism*.

[35] Wei L., Fraser J. L., Lu Z.-Y., Hu X., Yu S. P. (2012). Transplantation of hypoxia preconditioned bone marrow mesenchymal stem cells enhances angiogenesis and neurogenesis after cerebral ischemia in rats. *Neurobiology of Disease*.

[36] Ceradini D. J., Kulkarni A. R., Callaghan M. J. (2004). Progenitor cell trafficking is regulated by hypoxic gradients through HIF-1 induction of SDF-1. *Nature Medicine*.

[37] Laukkarinen J. M., van Acker G. J. D., Weiss E. R., Steer M. L., Perides G. (2007). A mouse model of acute biliary pancreatitis induced by retrograde pancreatic duct infusion of Na-taurocholate. *Gut*.

[38] Voronina S., Longbottom R., Sutton R., Petersen O. H., Tepikin A. (2002). Bile acids induce calcium signals in mouse pancreatic acinar cells: implications for bile-induced pancreatic pathology. *Journal of Physiology*.

[39] Hughes C. B., El-Din A. B. M., Kotb M., Gaber L. W., Gaber A. O. (1996). Calcium channel blockade inhibits release of TNF*α* and improves survival in a rat model of acute pancreatitis. *Pancreas*.

[40] Anzai Y. (2004). Superparamagnetic iron oxide nanoparticles: nodal metastases and beyond. *Topics in Magnetic Resonance Imaging*.

[41] Yano S., Kuroda S., Shichinohe H., Hida K., Iwasaki Y. (2005). Do bone marrow stromal cells proliferate after transplantation into mice cerebral infarct?—a double labeling study. *Brain Research*.

[42] Ittrich H., Peldschus K., Raabe N., Kaul M., Adam G. (2013). Superparamagnetic iron oxide nanoparticles in biomedicine: applications and developments in diagnostics and therapy. *RöFo*.

[43] Kijowski J., Baj-Krzyworzeka M., Majka M. (2001). The SDF-1-CXCR4 axis stimulates VEGF secretion and activates integrins but does not affect proliferation and survival in lymphohematopoietic cells. *Stem Cells*.

[44] Benest A. V., Salmon A. H., Wang W. (2006). VEGF and angiopoietin-1 stimulate different angiogenic phenotypes that combine to enhance functional neovascularization in adult tissue. *Microcirculation*.

[45] Nakamura T., Mizuno S. (2010). The discovery of hepatocyte growth factor (HGF) and its significance for cell biology, life sciences and clinical medicine. *Proceedings of the Japan Academy Series B: Physical and Biological Sciences*.

[46] Bai L., Lennon D. P., Caplan A. I. (2012). Hepatocyte growth factor mediates mesenchymal stem cell-induced recovery in multiple sclerosis models. *Nature Neuroscience*.

[47] Chen K., Mehta J. L., Li D., Joseph L., Joseph J. (2004). Transforming growth factor *β* receptor endoglin is expressed in cardiac fibroblasts and modulates profibrogenic actions of angiotensin II. *Circulation Research*.

[48] Chen M. M., Lam A., Abraham J. A., Schreiner G. F., Joly A. H. (2000). CTGF expression is induced by TGF-*β* in cardiac fibroblasts and cardiac myocytes: a potential role in heart fibrosis. *Journal of Molecular and Cellular Cardiology*.

[49] Grainger D. J. (2004). Transforming growth factor beta and atherosclerosis: so far, so good for the protective cytokine hypothesis. *Arteriosclerosis, Thrombosis, and Vascular Biology*.

[50] Dabek J., Kułach A., Monastyrska-Cup B., Gasior Z. (2006). Transforming growth factor *β* and cardiovascular diseases: the other facet of the “protective cytokine”. *Pharmacological Reports*.

